# The newborn heart GLAdly benefits from maternal milk

**DOI:** 10.20517/jca.2023.25

**Published:** 2023-07-26

**Authors:** Caitlyn E. Bowman, Zoltan Arany

**Affiliations:** 1 Cardiovascular Institute, Perelman School of Medicine, University of Pennsylvania, Philadelphia, PA 19104, USA.; 2 Biology Department, Williams College, Williamstown, MA 01267, USA.

The heart consumes fuel more avidly than any other organ in the body, a process necessary to sustain ceaseless systemic blood flow. The onset of cardiac beating occurs early during development, as fetal growth and development depend heavily on blood perfusion. *In utero* cardiac metabolism is largely fueled by carbohydrates readily provided by placental transport. During early postnatal life, however, the metabolic fuels that sustain the heart’s high energy demand switch largely to fatty acids, coincident with sudden access to high-fat milk and to oxygen^[1,2]^. This transition requires profound reprogramming of cardiac mitochondria via a combination of removal of old mitochondria via mitophagy and robust biogenesis of new mitochondria^[3]^.

How does the newborn heart know to undergo these changes? As in most biological contexts, the organism both anticipates the need and responds to environmental cues during the event. The latter include large hormonal shifts, increased access to oxygen, and dramatic changes in circulating metabolites and fuels, in part transmitted by maternal milk. A recent study by Paredes *et al*. now describes a new mechanism illustrating how a specific omega-6 fatty acid in mother’s milk activates a transcriptional program for mitochondrial fatty acid metabolism in the heart to promote this early postnatal metabolic adaptation^[4]^.

Paredes *et al*. originally set out to study the role of retinoid X receptor (RXR) receptors in the heart^[4]^. RXR receptors, *Rxra, Rxrb, and Rxrc* (poorly expressed in the heart), are ligand-activated nuclear receptors known to coordinate broad transcriptional programs and exhibit substantial functional redundancy^[4]^. Paredes *et al*., therefore, deleted both *Rxra* and *Rxrb* in myocardium by crossing floxed mice with *Nkx2.5-cre* mice, which promotes cardiac deletion from embryonic day E11.5 onward (EDKO mice)^[4]^. Remarkably, 80% of EDKO mice died in the first 24 h of postnatal life, and none survived past postnatal day 7. Echocardiographic analysis revealed severe cardiac contractile dysfunction.

What caused the cardiac failure? Gene expression profiling of postnatal day 0 (P0) hearts revealed transcriptional suppression in EDKO hearts of numerous genes involved in fatty acid oxidation and the mitochondrial carnitine shuttle. Further analyses of a subset of these genes, dubbed “mitochondrial fatty acid homeostasis” (mtFAH) gene cluster, revealed them to be normally induced in the heart during late gestation and further increased in early postnatal life, and this induction was prevented in the absence of *Rxra* and *Rxrb*. Epigenomic and transcriptomic analyses using ATAC-seq and ChIP-seq demonstrated that RXRs directly bind enhancers of mtFAH genes in the heart and facilitate chromatin remodeling consistent with transcriptional activation. Finally, evaluation of P0 cardiac mitochondria revealed decreased ATP production from palmitate, decreased long-chain acylcarnitine levels, and enhanced glycolytic flux and lactate production by EDKO heart mitochondria. From these data, the authors concluded that RXRs are critical mediators of cardiac metabolic maturation during late gestation and early postnatal life, reprogramming mitochondria to burn fatty acids.

What ligands are responsible for activating RXRs during this fetal-to-neonatal cardiac metabolic transition? Vitamin A is one well-known ligand of RXRs, and maternal milk contains vitamin A. However, maintaining dams on a vitamin A-deficient diet had no effect on P0 hearts, suggesting vitamin A was not the relevant ligand. In contrast, pups born to mothers on a fat-free diet did reveal reduced cardiac expression of mtFAH genes and impaired cardiac contractile function by echocardiography, akin to EDKO pups. Cross-fostering wildtype pups exposed to normal chow *in utero* to mothers on a fat-free diet had a similar effect, and all pups died within 48 h of birth. Together, these data suggested that one or more lipids in milk activate the cardiac RXR axis that promotes neonatal fuel switching. Paredes *et al*. next used lipidomic analysis of maternal milk to identify such lipids^[4]^. They identified omega-6 fatty acids as the most significantly downregulated lipid class in milk from mice on the fat-free diet.

Omega-6 fatty acids are essential fatty acids that cannot be synthesized by mammals and must be acquired from the diet (their relative absence in milk from dams fed a fat-free diet is, therefore, perhaps not surprising). Linoleic acid (LA; C18:2n-6) is the most abundant dietary essential omega-6 fatty acid. LA can be converted to γ-linolenic acid (GLA; C18:3n-6) by delta-6-desaturase, further converted to dihomo-γ-linolenic (DGLA; C20:3n-6), then arachidonic acid (AA; C20:4n-6), and from AA to the biosynthesis of numerous potent signaling molecules including prostaglandins, thromboxanes, and leukotrienes. LA and GLA were, in fact, already previously determined to bind RXRa and stabilize the heterodimerization that is required for transcriptional activation^[5,6]^. Paredes *et al*., therefore, first used neonatal cardiomyocytes and HL-1 cells to show that GLA is sufficient to induce the expression of mtFAH genes in cell culture^[4]^. More impressively, they next showed *in vivo* that supplementing GLA in maternal chow from mid-gestation onward was sufficient to rescue the postnatal lethality and the aberrant cardiac mtFAH gene signature seen in pups from mothers on a fat-free diet. Finally, they showed that this rescue by GLA required cardiac expression of RXRs, because it was not seen in EDKO mice. From these data, the authors conclude that GLA in maternal milk drives an RXR-dependent transcriptional maturation program required for postnatal viability.

The study by Paredes *et al*.^[4]^ is interesting and important, adding a new member to a growing but still small list of bioactive molecules in maternal milk that impact postnatal development^[7–9]^ and, importantly, providing a molecular explanation for its actions on the heart. The study does have important caveats. (1) Much is understandably made of the role of GLA in maternal milk, but the data presented more convincingly make an argument that GLA acts on cardiac RXRs *prenatally* to control cardiac maturation. Activation of the mtFAH gene signature and its suppression by RXR deletion is initiated before birth, and omega-6 fatty acids circulate abundantly in maternal plasma and can cross the placenta. GLA in the milk may, therefore, be continuing a program initiated during gestation; (2) It is also possible that GLA may require conversion of GLA to DGLA or AA to impact cardiac differentiation, although a direct effect by GLA is rendered more likely by the authors’ demonstration of direct binding of RXRa and GLA using surface plasmon resonance. Nevertheless, it is important to note that omega-6 fatty acids are important precursors to both pro- and anti-inflammatory molecules, and the observed cardiac failure may stem from such effects rather than metabolic alterations; (3) Finally, GLA and other omega-6 fatty acids likely have effects well beyond RXR activation. The fatty acids are, after all, essential fatty acids. For example, in the heart, the important mitochondrial lipid cardiolipin is almost entirely composed of LA acyl chains, and thus critically requires these essential fatty acids^[10]^. Depriving dams and pups of LA likely impacted mitochondrial membrane composition, and thus function, in the various conditions studied by Paredes *et al*.

How should we interpret this work in the context of human breastfeeding? The WHO and the U.S. Dietary Guidelines for Americans (2020–2025) strongly recommend exclusive breastfeeding for the first 6 months, based on increasingly strong evidence of the benefits of maternal milk. How these benefits are conferred remains incompletely understood, and the work of Paredes *et al*. suggests that omega-6 fatty acids may be critical to at least some of these benefits. One caveat when comparing rodents to humans, however, is the much lower adiposity in newborn rodents, rendering them especially vulnerable to postnatal essential fatty acid deprivation. Human neonates, in contrast, can mobilize omega-6 fatty acids from adipose depots established during fetal life^[11]^. Nevertheless, there is some evidence that infants fed breastmilk low in GLA - and perhaps exposed to lower GLA levels *in utero* - have lower growth rates, likely because their adipose stores of omega-6 fatty acids are insufficient^[12]^. Conversely, there is also some evidence that supplementation of essential fatty acids including GLA to preterm infants increased weight and length gain in the first months of life^[13]^. The work of Paredes *et al*. provides strong impetus to continue similar human trials, including assessment of outcomes beyond growth, such as cardiac function.
